# Age-related alterations in the modulation of intracortical inhibition during stopping of actions

**DOI:** 10.18632/aging.101741

**Published:** 2019-01-22

**Authors:** Lize Hermans, Celine Maes, Lisa Pauwels, Koen Cuypers, Kirstin-Friederike Heise, Stephan P. Swinnen, Inge Leunissen

**Affiliations:** 1 KU Leuven, Movement Control and Neuroplasticity Research Group, Department of Movement Sciences, Biomedical Sciences, Leuven, Belgium; 2 KU Leuven, Leuven Brain Institute (LBI), Leuven, Belgium

**Keywords:** transcranial magnetic stimulation, reactive inhibition, proactive inhibition, healthy aging, GABA, stop-signal

## Abstract

We investigated the effect of age on the ability to modulate GABA_A_-ergic and GABA_B_-ergic inhibitory activity during stopping of action (reactive inhibition) and preparation to stop (proactive inhibition). Twenty-five young and twenty-nine older adults performed an anticipated response version of the stop-signal task with varying levels of stop-signal probability. Paired-pulse transcranial magnetic stimulation was applied to left primary motor cortex to assess the modulation of GABA_A_-mediated short-interval intracortical inhibition (SICI) during stopping and GABA_B_-mediated long-interval intracortical inhibition (LICI) during the anticipation of a stop-signal. At the behavioral level, reactive inhibition was affected by aging as indicated by longer stop-signal reaction times in older compared to young adults. In contrast, proactive inhibition was preserved at older age as both groups slowed down their go response to a similar degree with increasing stop-signal probability. At the neural level, the amount of SICI was higher in successful stop relative to go trials in young but not in older adults. LICI at the start of the trial was modulated as a function of stop-signal probability in both young and older adults. Our results suggest that specifically the recruitment of GABA_A_-mediated intracortical inhibition during stopping of action is affected by aging.

## Introduction

The ability to inhibit an intended movement is a key component of cognitive control that allows flexible behavior in everyday life. Inhibition of motor responses is mediated by activity in a cortico-basal ganglia network whose output is thought to modulate the excitability of neurons within the primary motor cortex (M1) [1–4]. Whereas the direct pathway can excite motor cortex output via the striatum, the indirect and hyperdirect basal ganglia pathways can inhibit motor cortex excitability and support the suppression of undesired actions [5,6]. In accordance with this view, transcranial magnetic stimulation (TMS) studies show a decrease in corticospinal excitability (CSE) within M1 at 100-200 ms after the presentation of a stop-signal [7–9]. Notably, this suppression in excitability is supposedly accompanied by increased activation of GABAergic interneurons in M1 [7,10,11]. Intracortical interneurons receive and integrate input from cortical and subcortical structures [12], and can modulate the activity of corticospinal neurons via synaptic connections.

Different types of local intracortical inhibition in M1 can be investigated with paired-pulse TMS. Paired-pulse TMS protocols involve a conditioning stimulus (CS) that influences the size of the motor evoked potential (MEP) elicited by the subsequent test stimulus (TS). When the inter stimulus interval (ISI) is set between 1-3 ms or 50-200 ms, the CS will have an inhibitory effect on the size of the MEP elicited by the TS [13–16]. These phenomena refer to short-interval intracortical inhibition (SICI) and long-interval intracortical inhibition (LICI), respectively. SICI with an ISI of 2-3 ms is thought to reflect activity of GABA_A_ receptors [17,18], whereas LICI is thought to reflect activity of GABA_B_ receptors [19,20]. Specifically, the activity of the fast-acting GABA_A_ receptors has been found to increase in successful stop compared to go trials with the use of paired-pulse TMS [7].

Intracortical inhibitory processes may not only be recruited during the active cancellation of the motor response, but also proactively, i.e. in advance of preparing for upcoming stops. Evidence for this account has been provided by studies in which selective stop tasks were used [21–24]. When participants had foreknowledge about which effector might needed to be stopped, CSE of that effector was suppressed even before the stop-signal was presented [21,22]. Another way of assessing proactive inhibition is by manipulating the stop-signal probability. Research has shown that participants slow down their go response when the likelihood of an upcoming stop-signal increases; this is termed proactive response slowing [25–28]. In this respect, Cowie et al. [24] observed an increase in GABA_B_-mediated LICI in trials in which a stop-signal could occur compared to certain go trials. These results are consistent with the idea that proactive recruitment of intracortical inhibitory processes may occur under certain conditions.

The efficiency of motor inhibition has been shown to decrease as a function of age [29–32]. Emerging evidence indicates that the age-related motor inhibition impairments reflect a slower process of stopping a prepotent motor response (i.e. reactive inhibition), rather than an inability to anticipate upcoming stops (i.e. proactive inhibition) [33–35]. Yet, research into the neural substrates underlying these behavioral findings remains scarce. TMS research has shown that the ability to modulate GABAergic intracortical inhibition during motor tasks is diminished in older adults (for a review, see [36]). The effect of age on the modulation of SICI during motor inhibition has previously been investigated using a go/nogo task [37]. TMS was delivered in the period after the warning signal and the imperative signal. A release of inhibition was reported after the imperative signal, immediately before the onset of electromyography (EMG) activity in go trials in both young and older adults, and no changes in inhibition in nogo trials. It is possible however that participants in this study adopted a waiting strategy as response accuracy on the nogo trials was very high (on average 98% correct responses), suggesting that the nogo condition reflected the suppression of a response that was not yet initiated. Thus, it remains to be determined whether there are age-related differences in the modulation of intracortical inhibition during the cancellation of a prepotent response. Moreover, the role of inhibitory processes mediating proactive inhibition in older adults is yet to be established.

In the present study, we investigated the effect of age on the modulation of GABA_A_-mediated SICI and GABA_B_-mediated LICI during reactive and proactive inhibition, respectively. An anticipated response version of the stop-signal task with varying stop-signal probabilities was used. TMS was applied at two different time points: 1) 500 ms before the go response had to be made (i.e. early) to assess proactive recruitment of LICI, and 2) 150 ms after presentation of the stop-signal (i.e. late) in stop trials and at the same time point on go trials to assess recruitment of SICI during stopping. We hypothesized that 1) SICI would be higher in successful stop compared to go trials in young but not in older adults as reactive inhibition efficiency decreases with aging, 2) there would be proactive modulation of LICI at the early stimulation time point in young adults, which might be preserved in older adults because behavioral research demonstrated that proactive inhibition remains intact with aging.

## RESULTS

Two older adults did not complete the TMS sessions due to high TS intensity (≥ 80 maximum stimulator output, N = 1) or high background EMG (N = 1). The SICI data of one older adult was excluded due to artifacts in the EMG data. Lastly, SICI data of one young adult was excluded due to high background EMG. Hence, a total of N = 26 of the older and N = 24 of the younger of the SICI data sets, and a total of N = 27 of the older and N = 25 of the younger LICI data sets were entered in the statistical analyses.

### Stop-signal task

Table 1 shows stop-signal task performance during the behavioral assessment at the start of each session, and during TMS.

**Table 1:** Summary of behavioral results.

			SICI			LICI		
no-TMS			Young	Older		Young	Older	
Early response (%)			0 ± 0	0.07 ± 0.27		0 ± 0	0.23 ± 0.48	
No response (%)			0.49 ± 1.25	0.35 ± 0.78		0.62 ± 1.71	0.55 ± 1.06	
P(inhibit) %			53.2 ± 1.7	52.7 ± 1.6		52.2 ± 0.8	52.4 ± 1.7	
SSRT (ms)			192 ± 15	207 ± 19		195 ± 13	205 ± 23	
GoRT (ms)	0%		801 ± 11	808 ± 18		803 ± 12	806 ± 24	
	20%		814 ± 11	820 ± 15		813 ± 10	820 ± 27	
	40%		822 ± 13	838 ± 26		825 ± 13	831 ± 28	
TMS								
Stop time (ms)			547 ± 17	534 ± 27		545 ± 18	536 ± 27	
P(inhibit) %			70.6 ± 7.6	67.8 ± 11.6		70.2 ± 7.6	65.1 ± 11.6	

Data are reported as mean ± SD. Go response times (GoRTs) are reported from bar fill onset, the target was situated 800ms from bar fill onset. Early responses are GoRTs < 400 ms, whereas no responses are GoRTs > 1280 ms. Percentage of early responses and no responses were averaged across the three stop-signal probability conditions. LICI: long-interval intracortical inhibition, SICI: short-interval intracortical inhibition, SSRT: stop-signal reaction time, TMS: transcranial magnetic stimulation.

### *Behavioral assessment pre-TMS*

The P(inhibit) was close to 50% and did not differ between young and older adults (*F*(1, 48) = 0.325, *p* = .571, η_p_^2^ = .007), allowing reliable estimation of the stop-signal reaction time (SSRT) with the integration method. There was a small (<1%), yet significant difference in P(inhibit) between the two sessions (*F*(1, 48) = 4.406, *p* = .041, η_p_^2^ = .084). The group by session interaction was not significant (*F*(1, 48) = 1.413, *p* = .240, η_p_^2^ = .029).

Older adults had significantly longer SSRTs compared to young adults (*F*(1, 48) = 8.854, *p* = .005, η_p_^2^ = .156). There was no main effect of session (*F*(1, 48) = 0.147, *p* = .703, η_p_^2^ = .003), and no interaction between age group and session (*F*(1, 48) = 0.755, *p* = .389, η_p_^2^ = .015). There was a trend for shorter go response times (GoRTs) in young compared to older adults (*F*(1, 48) = 3.607, *p* = .064, η_p_^2^ = .070). Furthermore, GoRTs significantly increased as a function of stop-signal probability (SSP, *F*(1.736, 83.338) = 93.037, *p* < .001, η_p_^2^ = .660) to a similar degree in young and older adults (*F*(1.736, 83.338) = 1.362, *p* = .260, η_p_^2^ = .028). Post hoc comparisons indicated that GoRTs significantly increased from 0% to 20% SSP (*p* < .001) and from 20% to 40% SSP (*p* < .001). All other main effects and interactions failed to reach significance (all *F* ≤ 1.865, all *p* ≥ .160).

### *Behavioral assessment during TMS*

There was a trend for earlier participant-specific stop times during TMS for older compared to young adults (*F*(1, 48) = 3.795, *p* = .057, η_p_^2^ = .073). There was no session effect (*F*(1, 48) = 0.003, *p* = .955, η_p_^2^ = .000) and no interaction between age group and session (*F*(1, 48) = 0.515, *p* = .477, η_p_^2^ = .011). The P(inhibit) during TMS ranged on average between 65-70% and did not differ between age groups (*F*(1, 48) = 2.619, *p* = .112, η_p_^2^ = .052). There was no effect of session (*F*(1, 48) = 1.236, *p* = .272, η_p_^2^ = .025) and no interaction between age group and session (*F*(1, 48) = 0.653, *p* = .423, η_p_^2^ = .013).

### TMS

### *TMS intensities and motor threshold*

TMS intensities and motor threshold are summarized in Table 2. The 2-way ANOVA_RM_ revealed that the TS intensity was significantly higher in the SICI compared to LICI session (*F*(1, 48) = 6.289, *p* = .016, η_p_^2^ = .116). There was no significant difference in the TS intensity between age groups (*F*(1, 48) = 0.060, *p* = .807, η_p_^2^ = .001) and no interaction between age group and session (*F*(1, 48) = 0.003, *p* = .958, η_p_^2^ = .000). There were no group differences in CS intensity (*t*(48) = 0.006, *p* = .995) or tMT (*t*(43.687) = -0.344, *p* = .732).

**Table 2:** TMS intensities, resting corticospinal excitability and intracortical inhibition.

	SICI			LICI		
	Young	Older		Young	Older	
TS intensity (% MSO)	60 ± 1.8	61 ± 2.2		59 ± 1.8	60 ± 1.9	
CS intensity (% MSO)	40 ± 1.2	40 ± 1.6		/	/	
tMT (% MSO)	50 ± 1.4	51 ± 2.2		/	/	
Unconditioned MEP in rest (mV)	1.2 ± 0.2	0.9 ± 0.08		0.8 ± 0.08	0.9 ± 0.1	
Inhibition in rest (%)	71 ± 3.5	52 ± 8.0		83 ± 4.4	47 ± 10.8	

Data are reported as mean ± SEM. Inhibition was calculated as follows: [1 – (MEPCS + TS / MEPTS)] * 100. CS: conditioning stimulus, LICI: long-interval intracortical inhibition, MSO: maximum stimulator output, SICI: short-interval intracortical inhibition, tMT: task motor threshold, TS: test stimulus.

### *Resting-state measures of CSE, SICI and LICI*

CSE (represented by the unconditioned motor evoked potential (MEP)) in rest did not differ between age groups (Table 2) (*F*(1, 48) = 1.340, *p* = .253, η_p_^2^ = .027) or sessions (*F*(1, 48) = 2.204, *p* = .144, η_p_^2^ = .044). There was also no interaction between age group and session (*F*(1, 48) = 1.849, *p* = .180, η_p_^2^ = .037). There was an age effect on SICI in rest (*t*(13.533) = 2.101, *p* = .043) and LICI in rest (*U* = 212, *p* = .022), with lower inhibition in older compared to young adults.

### *Modulation of CSE and SICI during reactive inhibition*

**Figure 1:**
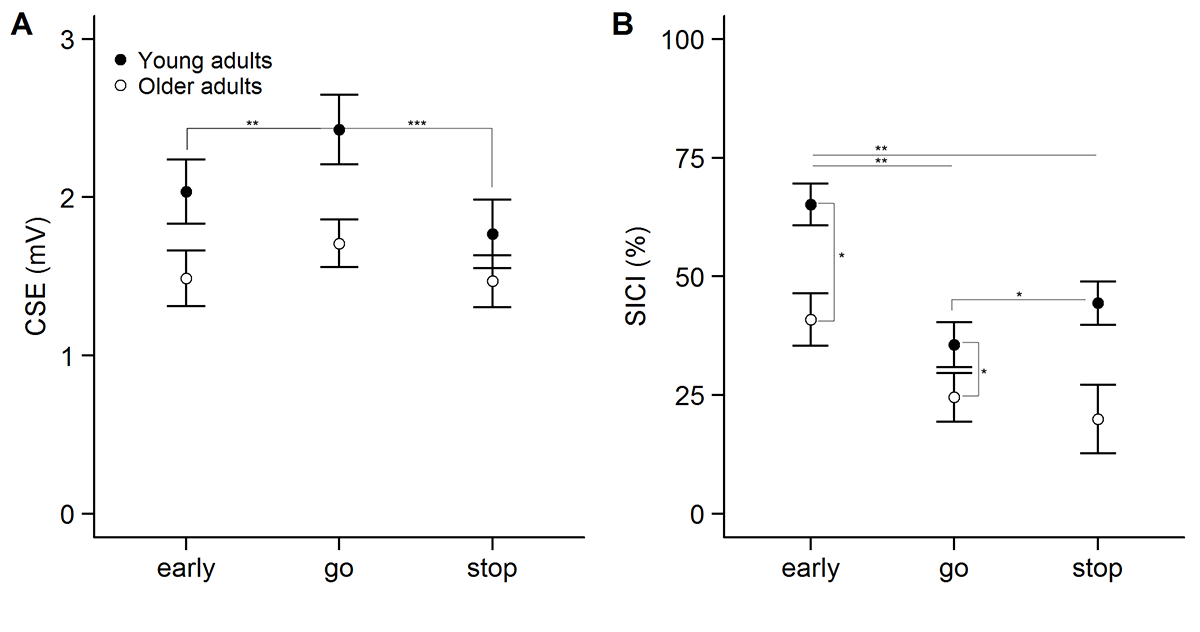
Modulation of corticospinal excitability (CSE, panel **A**) and short-interval intracortical inhibition (SICI, panel **B**) across the three conditions in the SICI session. Statistically significant differences in the interaction term are described in the main text. CSE is measured as unconditioned motor evoked potential (MEP) amplitude. Inhibition was calculated as follows: [1 – (MEP_CS + TS_ / MEP_TS_)] * 100. Error bars represent standard error of mean. ***p<0.001, **p<0.01, *p<0.05, ^p<0.1.

Changes in CSE across the three conditions in the SICI session are shown in Figure 1A. On average, a total of 30 ± 11 trials per condition were included to assess CSE in the analysis. The 2-way ANOVA_RM_ indicated that there was a main effect of age group (*F*(1, 48) = 4.307, *p* = .043, η_p_^2^ = .082) in which CSE was found to be lower in older versus young adults. Furthermore, a main effect of condition (*F*(1.714, 82.268) = 18.867, *p* < .001, η_p_^2^ = .282) and a significant interaction between age group and condition (*F*(1.714, 82.268) = 3.984, *p* = .028, η_p_^2^ = .077) was observed. Post-hoc comparisons indicated that MEP amplitudes were reduced in early compared to go (*p* = .006), and stop compared to go (*p* < .001) in young adults. The difference between early and stop was not significant (*p* = .141). In older adults, there were no differences in MEP amplitude between conditions (all *p* ≥ .199). CSE did not significantly differ between young and older adults in any of the trial types (all *p* ≥ .090).

**Figure 2:**
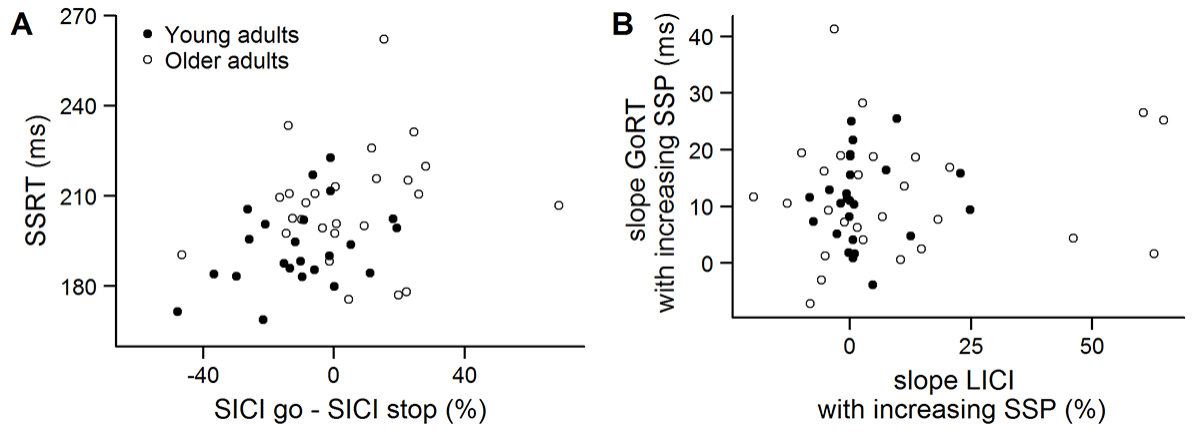
(**A**) Relation between the stop-signal reaction time (SSRT) in the short-interval intracortical inhibition (SICI) session and the difference in SICI between go and stop conditions in young (black) and older adults (white). (**B**) Relation between the slope of the go response time (GoRT) and the slope of long-interval intracortical inhibition (LICI) in the early condition (300 ms after trial onset, untransformed data). The slope was calculated as a function of stop-signal probability.

The modulation of SICI across the three conditions is shown in Figure 1B. On average, a total of 30 ± 11 unconditioned trials and a total of 30 ± 11 conditioned trials per condition were used to calculate SICI in the analysis. The 2-way ANOVA_RM_ indicated that there was a significant main effect of age group (*F*(1, 47) = 6.838, *p* = .012, η_p_^2^ = .127), with higher inhibition in young compared to older adults. Furthermore, a main effect of condition (*F*(2, 94) = 13.685, *p* < .001, η_p_^2^ = .226) and a significant interaction between age group and condition (*F*(2, 94) = 3.296, *p* = .041, η_p_^2^ = .066) was observed. Post-hoc comparisons showed that inhibition was higher in early compared to stop and go in both young and older adults (all *p* ≤ .003). SICI was significantly higher in young compared to older adults in the early (*p* = .026) and stop condition (*p* = .024), but not in the go condition (*p* = .694). Finally, planned comparisons indicated that there was more inhibition in stop compared to go in young (*p* = .036) but not in older adults (*p* = .231). These results suggest that the recruitment of SICI during reactive inhibition was affected in older adults. In spite of that, there was no significant correlation between the modulation of SICI (SICI go – SICI stop) and SSRT in young (r = .108, *p* = .615) or older adults (r = .151, *p* = .461) (Figure 2A).

### *Modulation of CSE and LICI as a function of stop-signal probability*

**Figure 3:**
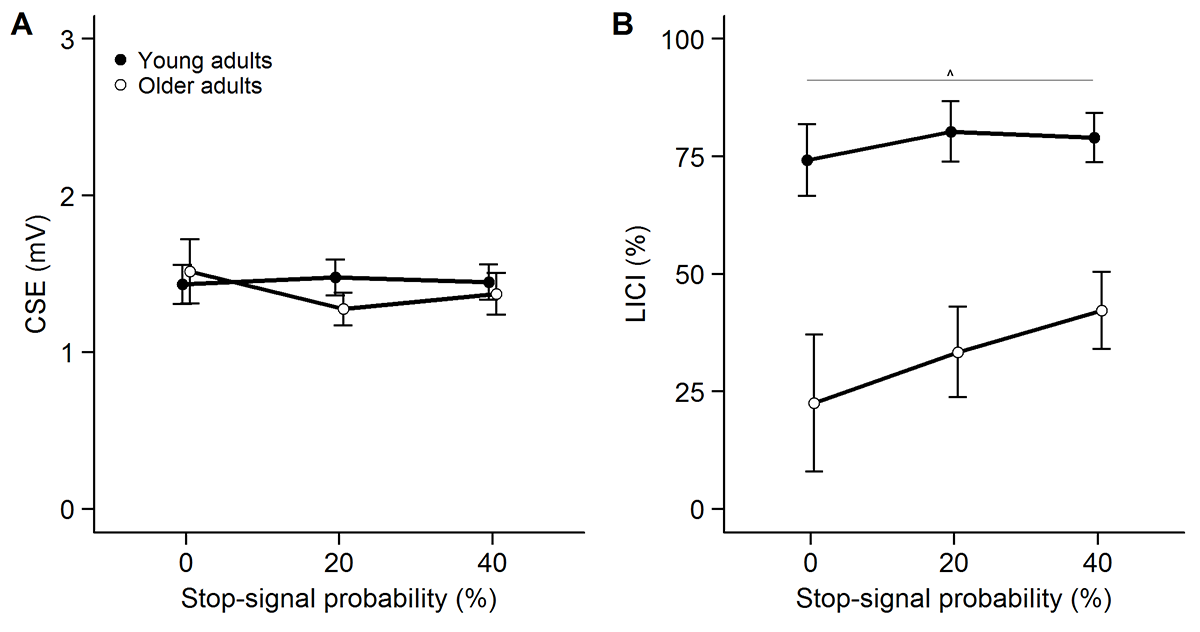
Modulation of corticospinal excitability (CSE, panel **A**) and long-interval intracortical inhibition (LICI, panel **B**) as a function of stop-signal probability (SSP) in the LICI session. CSE is measured as unconditioned motor evoked potential (MEP) amplitude. Inhibition was calculated as follows: [1 – (MEP_CS + TS_ / MEP_TS_)] * 100. Untransformed LICI values are presented. Error bars represent standard error of mean. ***p<0.001, **p<0.01, *p<0.05, ^p<0.1.

The modulation of CSE as a function of SSP in the early condition in the LICI session (Figure 3A) was investigated with a 2-way ANOVA_RM_. On average, a total of 18 ± 4 trials per condition were included to assess CSE in the analysis. There was no effect of age group (*F*(1, 50) = 0.132, *p* = .718, η_p_^2^ = .003), no effect of SSP (*F*(1.556, 77.805) = 1.113, *p* = .321, η_p_^2^ = .022) and no interaction between age group and SSP (*F*(1.556, 77.805) = 2.300, *p* = .119, η_p_^2^ = .044).

On average, a total of 18 ± 4 unconditioned trials and a total of 18 ± 4 conditioned trials per condition were used to calculate LICI in the early condition. Analysis of the LICI data showed that inhibition was significantly higher in young compared to older adults (Figure 3B) (*F*(1, 49) = 20.153, *p* < .001, η_p_^2^ = .291). Furthermore, there was a main effect of SSP on LICI (*F*(1.787, 87.539) = 4.195, *p* = .022, η_p_^2^ = .079). Post-hoc tests revealed a trend for higher inhibition in the 40% SSP condition compared to the 0% SSP condition (*p* = .083). The interaction between group and SSP did not reach significance (*F*(1.787, 87.539) = 2.089, *p* = .135, η_p_^2^ = .041). Furthermore, there was no significant correlation between the slope of LICI and the slope of GoRTs in young (LICI: 2 ± 8, GoRT: 11 ± 8; *r_S_* = .028, *p* = .895) or older adults (LICI: 10 ± 23, GoRT: 12 ± 11; *r_S_* = .087, *p* = .665) (Figure 2B).

## DISCUSSION

We investigated the effect of age on the modulation of GABA_A_-mediated versus GABA_B_-mediated neurotransmission during reactive and proactive inhibitory control. At the behavioral level, we found that reactive but not proactive inhibition was affected by aging. Results of the paired-pulse TMS measurements showed that GABA_A_-mediated inhibition, indicated by SICI, was higher during stopping compared to go trials in young, but not in older adults. Furthermore, GABA_B_-mediated inhibition, as indicated by LICI at the start of the trial, was modulated as a function of stop-signal probability in both young and older adults. These results suggest that specifically the recruitment of GABA_A_-ergic inhibitory activity during the successful cancellation of a prepotent motor response is altered by aging.

### Intracortical inhibition during action initiation and cancellation

CSE increased from the beginning of the trial (i.e. 300 ms after trial onset) to just before go response onset, corroborating previous findings [7,38]. At the same time, there was a release of GABA_A_-mediated inhibition immediately before the anticipated response had to be made, as indicated by a decrease in SICI from the beginning of the trial to the late stimulation point. Previous research indicates that the release of intracortical inhibition in contralateral M1 might even occur prior to the increase in excitability [39,40]. It is postulated that GABAergic inhibition serves as a brake which needs to be removed for the execution of a motor response [39–41]. In contrast to previous findings [42], the release of GABA_A_-ergic inhibition before movement onset was also evident in older adults. A possible explanation for this discrepancy might be that participants in the present study had to make timed instead of speeded responses. More specifically, they had to respond when a filling bar crossed a horizontal target line at 800 ms from onset. These visual cues made it possible to precisely anticipate the go response, which might be less demanding than responding as fast as possible, and regulation of the system might be preserved under these conditions.

A release of GABA_A_-mediated inhibition was also observed during the successful cancellation of the motor response compared to the early time point. Previous findings with respect to the release of inhibition during successful stop trials compared to the start of the trial are contradictory. Whereas Coxon et al. [7] found comparable levels of SICI in the beginning of the trial and during stopping, a more recent study investigating motor inhibition in healthy controls and patients with schizophrenia [43] revealed that SICI in the healthy controls was modulated in a similar manner as in the present experiment. Possibly, the release of SICI during stopping reflects the release of the brake prior to voluntary movement [41], suggesting that participants were indeed preparing to respond and did not adopt a waiting strategy. Importantly, however, the amount of SICI was higher in successful stop than go trials in young adults, suggesting that GABA_A_-mediated inhibition is recruited more during stopping compared to the same time point in go trials and corroborating previous findings [7]. Taken together, these results provide evidence for the involvement of GABA_A_-mediated neurotransmission in the initiation and successful cancellation of movements.

As expected, our behavioral results show that older adults had significantly longer SSRTs compared to young adults, indicative of poorer reactive inhibition [29–31,33–35]. At the neural level, the amount of SICI did not differ between stop and go in older adults like it did in the young adults. The capacity to modulate GABA_A_-ergic inhibition during outright stopping thus seems to be affected by aging, possibly resulting in a slower stop process. Nonetheless, we did not observe a relationship between the difference in SICI between successful stop and go trials, and the duration of the stop process. This indicates that GABA_A_-mediated intracortical inhibition is likely not the sole mechanism responsible for the suppression of the motor response. Another indication for this assumption is that SICI was higher in the beginning of the trial compared to stop, while CSE was similar in both conditions. Further research into the mechanisms involved in successful stopping is warranted.

### Intracortical inhibition as a function of stop-signal probability

Proactive go response slowing was investigated by using three different stop-signal probabilities (0%, 20% and 40%) which were signaled by the color of the bar. Analysis of go response times showed that the degree of go response slowing with increasing stop-signal probability was comparable between young and older adults. In line with previous findings [33–35,44], these results suggest that proactive go response slowing is not affected by aging.

We investigated whether GABA_B_-ergic intracortical inhibition would be modulated as a function of stop-signal probability by stimulating early in the trial (300 ms after trial onset) before occurrence of the stop-signal or initiation of the go response, when there was 0%, 20% and 40% chance of stopping. The results showed that GABA_B_-mediated inhibition, as indicated by LICI, did change depending on the probability of upcoming stops. More specifically, a trend for an increase in LICI from 0% to 40% was observed. These results are in line with the findings of Cowie et al. [24]. They stimulated right M1 during a bimanual stop-signal anticipation task in which either part of the response or the whole response should be cancelled without foreknowledge. The increase in LICI during the foreperiod when stop trials were introduced was explained as a ‘tonic inhibitory process’ which raises the threshold for responding [24,38,45]. Due to the higher threshold, a further increase in facilitation is needed to reinitiate a subcomponent of the suppressed movement, causing delays in the ensuing component [23,24]. Yet, recently Cirillo et al. [23] found no difference in LICI after forewarning which effector (might) have to be stopped. Instead, it was SICI that was significantly modulated by the cues, in the sense that SICI was reduced when it was certain or very likely that the contralateral hand had to respond. Their suggestion is that LICI might be used to set general inhibitory tone according to task context and not cue information, whereas SICI might be modulated proactively with response certainty to optimize task performance. The increase in LICI in the present study supports the idea that there is a rise in the threshold for responding when there is more chance of stopping. This could be set at the start of the task blocks, however, despite the absence of a significant group by stop-signal probability interaction, the pattern of results suggest that older adults might modulate LICI on trial by trial basis given that LICI in the 40% SSP trials seems higher than in the 20% trials. This notion could fit with the observation that older adults tend to take a more cautious response strategy and thus exert more proactive control [46,47]. It should be noted however, that no correlations with behavior were found.

CSE did not change as a function of stop-signal probability. Previous studies using a 2-choice stop-signal task, showed that the excitability of the cortical representation of an effector is proactively suppressed when forewarning signals that this effector might need to be stopped [21,22]. Contrarily, Cowie et al. [24] found an increase in CSE when stopping as more likely, and Cirillo et al. [23] found no difference in CSE forewarning. It seems like CSE is modulated differently in the anticipation task compared to a 2-choice reaction task. Possibly CSE increases, or is not suppressed in the anticipation task to counteract rise in tonic inhibition to keep go responses on target [24].

### Overall age effects

Age-related differences in GABA_A_- and GABA_B_-mediated inhibition were observed in rest as well as during the task, as indicated by SICI and LICI. While studies on resting-state GABAergic inhibition in aging reported mixed results, most studies on task-related GABAergic inhibition reported age-related alterations [36]. Our findings add to a growing body of evidence highlighting age-related alterations to the GABA system that become particularly apparent during motor performance.

The observed lower inhibition in older adults could reflect a compensatory mechanism [42,48] to maintain adequate performance. This seems to be successful under low task demands such as when making timed responses. However, when older adults are under high temporal pressure, i.e. when an action needs to be canceled in response to an external cue, the modulatory capacity of the GABA_A_-ergic system is diminished, and performance becomes degraded.

## Conclusion

In summary, the current data show that older adults are able to proactively slow down their responses in anticipation of upcoming stops. Nevertheless, they demonstrate poorer performance compared to young adults when they reactively need to suppress a prepotent action. At the neural level, we find that GABA_A_-mediated SICI during action cancellation is modulated differently for older compared to young adults. These results reinforce the idea that measures of GABA-mediated intracortical inhibition can contribute to our understanding of age-related deficits in cancelling prepotent motor responses.

## METHODS

### Participants

A total of 25 young (23 ± 4 years, 12 women) and 29 older healthy adults (68 ± 4 years, 13 women) participated in this study. All participants were right-handed according to the Oldfield Handedness scale (91.7 ± 12.3, range = 57.1 – 100) and reported no history of neurological impairments. The experiment was approved by the local ethical committee of KU Leuven (protocol number: s58333) and all participants gave written informed consent.

### Procedure

**Figure 4:**
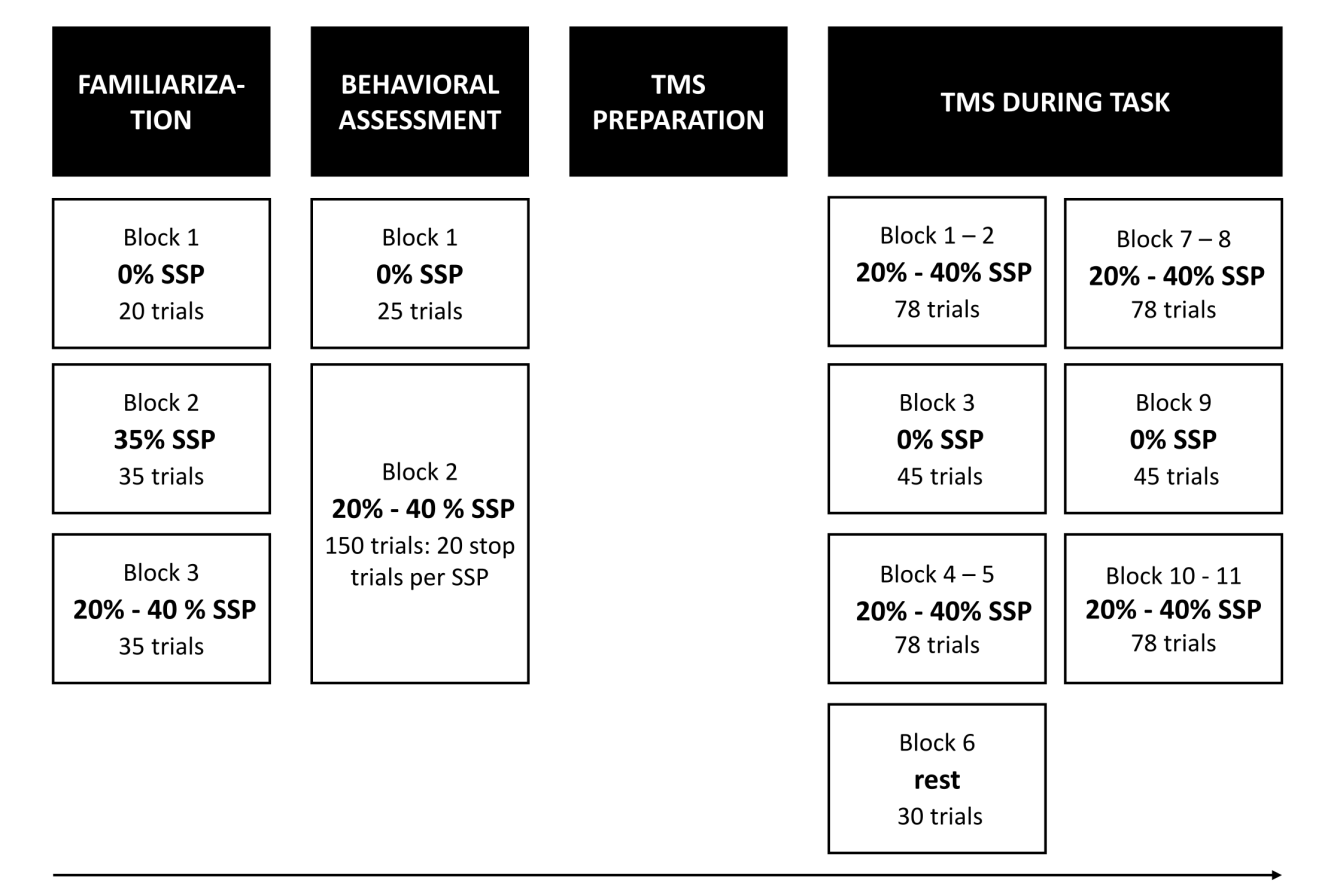
**Experimental procedure.** At the start of the session, participants first practiced the task. Immediately after familiarization, behavioral reactive and proactive inhibition was assessed (behavioral assessment). Next, TMS was prepared and delivered during the task. In total, there were 10 TMS task blocks and 1 TMS rest block, which were presented in the same order across participants. There were two different types of task blocks: one with certain-go trials (i.e. stop-signal probability [SSP] = 0%) and one with uncertain-go trials (i.e. SSP = 20 and 40%). During the rest block, resting measures of corticospinal excitability and intracortical inhibition were assessed.

SICI and LICI assessments were performed in two different sessions on separate days with at least 48h between sessions (counterbalanced, average # days between sessions: 12 ± 16). The procedure of the experiment was identical for the SICI and the LICI session (Figure 4).

### *Behavioral assessment*

**Figure 5:**
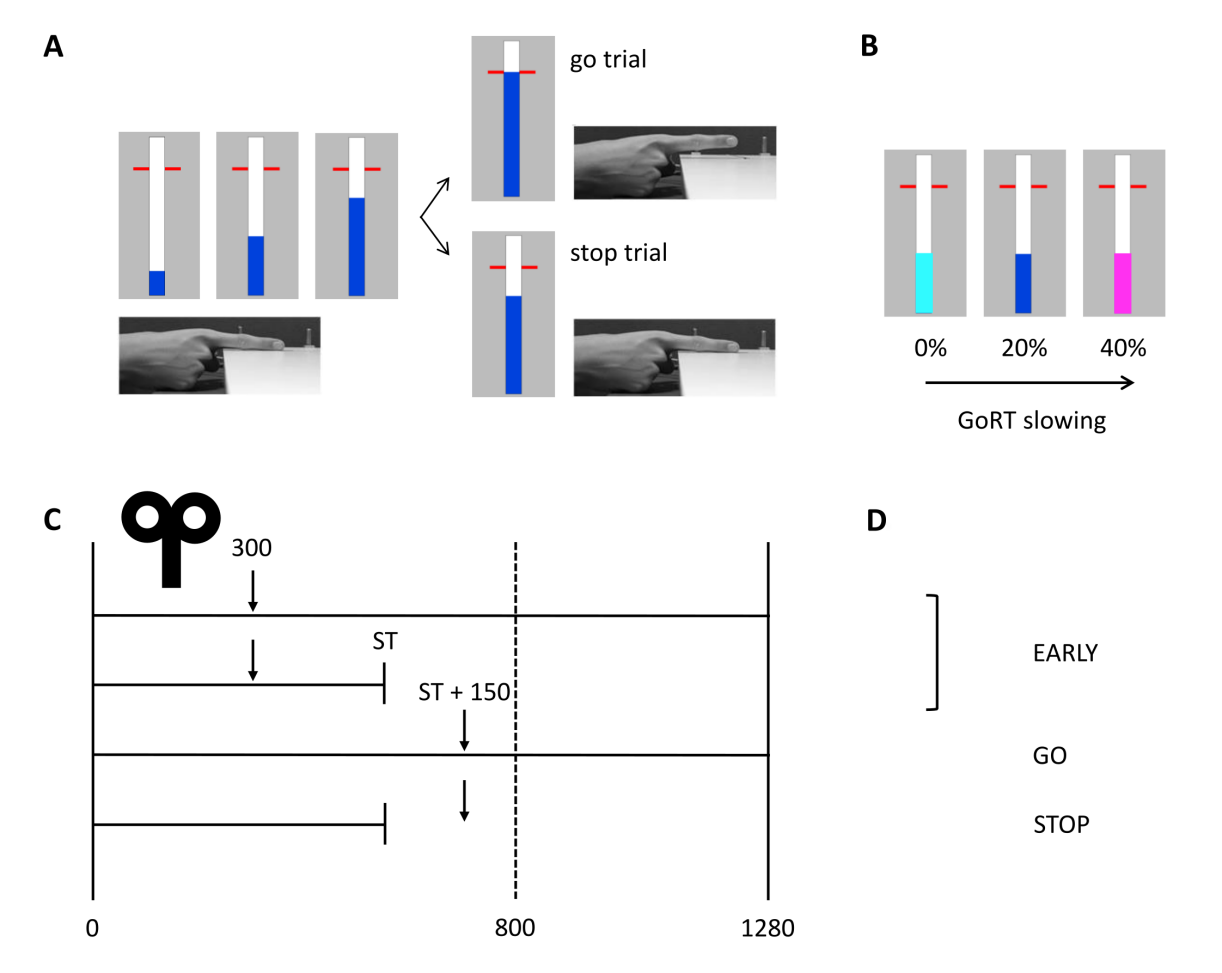
**Anticipated response version of the stop-signal paradigm with timing of TMS and conditions of interest.** (**A**) In go trials, participants had to stop the bar as close as possible to the horizontal target at 800 ms from trial onset. The bar could be stopped by releasing the right index finger from the switch. In stop trials, the bar stopped automatically and participants had to cancel the movement of lifting their finger. (**B**) Proactive inhibition was assessed by manipulating the stop-signal probability. The color of the bar indicated the probability of stops (light blue: 0%, blue: 20%, magenta: 40%). (**C**) The solid vertical lines represent the start (0 ms) and finish time (1280 ms) of the trial. The target is indicated by the vertical dashed line at 800 ms from trial onset. The TMS test stimulus was delivered at 300 ms from trial onset (early) or 150 ms after the participant-specific stop time (late) in go and stop trials. The stop time was determined for each participant separately and was based on task performance without TMS. (**D**) Three different conditions were included in the statistical analyses: early, stop and go. GoRT: go response time, ST: stop time.

An anticipated response version of the stop-signal task was used with different stop-signal probabilities to assess reactive and proactive inhibition (Figure 5A-B) [7,27,49]. Participants were seated on a chair and had to place their right hand on the table with their index finger on a switch. The switch was pressed when the finger was resting, requiring no active force production. On a computer screen (refresh rate = 60 Hz), a bar started to fill at a constant velocity and participants were instructed to stop the bar as close as possible to the horizontal target line (800 ms from trial onset) by releasing the switch, i.e. lifting their index finger (go trials). The color of the target line changed after each go trial providing feedback about task performance. The colors represent the absolute timing difference relative to the target: green: < 20 ms, yellow: < 40 ms, orange: < 60 ms and red: > 60 ms. In stop trials, the bar would automatically stop before reaching the target line and participants were instructed to cancel the movement of releasing the switch, i.e. not lift their finger and thus keep the switch pressed. A staircasing algorithm was used to ensure an equal number of successful and unsuccessful stop trials (i.e. P(inhibit) ≈ 50%). More specifically, the time point on which the bar automatically stopped filling on stop trials (i.e. stop time) was increased with 33 ms when stopping was successful and decreased with 33 ms when stopping was unsuccessful. To assess proactive inhibition, three SSPs were used (0%, 20% and 40%) (Figure 5B). The color of the bar indicated the probability of stops (light blue bar: 0%, blue bar: 20%, magenta bar: 40%).

At the beginning of each session, all participants practiced the task (Familiarization, Figure 4). We instructed participants to aim for green or at least yellow lines after go trials to reinforce go task performance. The participants were told that it would not be possible to cancel the movement of lifting their finger on all stop trials. Finally, participants were instructed that the probability of stops was lower when the bar was dark blue compared to magenta and that only go trials would occur when the bar was light blue. After the practice blocks, participants were asked to describe the meaning of the three colors to make sure that the instructions were understood correctly.

Next, they completed one block with 0% SSP (25 go trials) and one block in which 20% and 40% SSP were randomly varied (150 trials). The number of stop trials was matched between the two SSPs resulting in 80 go and 20 stop trials for the 20% condition and 30 go and 20 stop trials for the 40% condition (Figure 4). Inter trial interval was set at 3.25 ms and the bar was reset to empty 1.28 s after trial onset.

### *TMS preparation and EMG*

SICI and LICI were performed with two Magstim 200 units connected through a BiStim module (Magstim Company, Dyfed, UK). A figure-of-eight-coil (70 mm outer diameter) was placed over the motor hotspot of the right first dorsal interosseous (FDI) with the handle pointing posteriorly at an approximately 45° angle to the midsagittal plane. EMG activity was recorded within the right FDI with EMG surface electrodes (Ag/AgCl) using a belly-tendon montage (Bagnoli-16 EMG system, Delsys Inc., Boston, USA). EMG signals were amplified (gain = 1000), bandpass filtered (20-2000 Hz) and 50 Hz noise was eliminated (Humbug, Quest Scientific, North Vancouver, Canada). The signals were sampled at 5000 Hz and stored for offline analysis (CED Signal Version 4.03, Cambridge Electronic Design, UK).

An orthogonal 1x1 cm coordinate system was marked on a swimming cap with references to the left and right external auditory meatus, occiput and vertex. The motor hotspot was defined as the location from which five consecutive TMS pulses produced the highest mean MEP within relaxed right FDI. The motor hotspot was marked on the cap and captured with a neuronavigation system (Visor 2, ANT Neuro, the Netherlands) with the standard MNI template to ensure optimal positioning of the magnetic coil during the experiment.

For SICI, an ISI of 3 ms was used. The TS intensity was set to elicit an MEP of approximately 1 mV when the finger was on the switch. To determine the CS intensity, we first measured the task motor threshold (tMT). This threshold was defined as the minimum intensity required to elicit four out of eight MEPs when the finger was on the switch [24]. The CS intensity for SICI was then set to 90% tMT and systematically lowered with 2% until relative inhibition was on average 50% (average of five consecutive paired-pulses).

For LICI, an ISI of 100 ms was used. The TS and CS intensities were set to elicit an MEP of approximately 1 mV when the finger was on the switch.

### *TMS during task*

Participants performed ten task blocks per session while TMS was applied (see Figure 4). Consistent with Cowie et al. [24], the go only trials (i.e. SSP = 0%) were presented in separate blocks. The 20 and 40% SSP conditions were randomly varied within the other eight task blocks. In the TMS task blocks, the TS was randomly applied at two different time points: early (i.e. 300 ms after trial onset / 500 ms before the target) or late (i.e. participant-specific stop time + 150 ms) (Figure 5C-D). The late stimulation point was chosen because active inhibition is expected 150 ms after presentation of the stop-signal [7]. The participant-specific stop time was previously determined from the no-TMS task block performed earlier with the following formula: 800 ms – [mean(GoRT) – mean(stop time)] – 50 ms. The average response time in go trials (GoRT) was defined as the time between trial onset and lifting the finger from the switch, and thus a GoRT of 800 ms reflects a response on the target. By using a participant-specific stop time, the degree of stopping difficulty is comparable across participants. By subtracting 50 ms, the bar stops earlier and the P(inhibit) during TMS increases, resulting in more successful stop trials and thus more trials that can be included in the analysis. Furthermore, it reduces the chance that the TMS pulse is applied after EMG onset in go trials. The participant-specific stop time during TMS was kept constant. To keep the participant alert, catch trials were introduced in which the bar stopped 125 ms after the participant-specific stop time and thus closer to the target. Consequently, stopping was more difficult in these trials.

In total, there were 714 trials (see Figure 4 for number of trials per block). For the early stimulation time point, 20 single-pulse and 20 paired-pulse trials were administered per SSP condition. For the late stimulation time point, 24 single-pulse and 24 paired-pulse trials were delivered in the 0% SSP condition, and 56 single-pulse and 56 paired-pulse in both the 20% and 40% SSP conditions. Out of these 56 trials, 28 were stop trials. More trials were included in the late compared to the early stimulation time point because there is a higher chance that former trials will be excluded due to EMG onset. There were 2, 232 and 54 non-stimulated go trials in the 0%, 20% and 40% SSP condition, respectively. Finally, there were 16 non-stimulated stop trials in the 20% SSP condition and 18 non-stimulated stop trials in the 40% SSP condition. These non-stimulated stop trials were the catch trials in which the bar stopped 125 ms after the participant-specific stop time. The trial duration was 3.25, 4 or 4.45 s depending on the time point of stimulation in the previous and current trial, such that there was at least 4 s between two consecutive TS pulses.

### *TMS during rest*

Resting-measures of CSE and intracortical inhibition were assessed during the rest block, which was always performed halfway through the experiment (i.e. block 6, Figure 4). Participants were instructed to look at a white fixation cross presented in the center of a black screen while their finger was resting on the switch. A total of 15 single-pulse and 15 paired-pulse trials were conducted in a random order at the same intensities as described above.

### Analysis

### *Behavioral analysis*

Behavioral analysis was performed on baseline performance prior to TMS. GoRTs shorter than 400 ms (i.e. early response) or longer than 1280 ms (i.e. no response) were considered errors and were removed from analysis. The average GoRT for each SSP was calculated. The amount of response slowing (GoRT) with increasing SSP served as a measure of proactive inhibition. The percentage of successful stop trials (P(inhibit)) was calculated across the 20 and 40% SSP. Next, the stop-signal reaction time (SSRT) was calculated across the 20% and 40% SSP conditions with the integration method [50]. More specifically, the number of failed stop trials was divided by the total number of stop trials to get P(respond). GoRTs were sorted in ascending order and the nth GoRT was obtained where n equals the number of go trials multiplied by P(respond) [50]. The SSRT was estimated by subtracting the average stop time from the nth GoRT. The SSRT reflects the speed of stopping and served as a measure of reactive inhibition.

Variables of interest for the task during TMS were the percentage of successful stop trials (i.e. P(inhibit)) and the participant-specific stop time. GoRTs and SSRT during TMS were not investigated since TMS may influence response times.

### *EMG data processing and analysis*

All trials were visually inspected and excluded if there was activity in the period between the TS and the onset of the MEP. The root mean square (RMS) of the EMG signal 50 ms prior to the first TMS pulse was calculated for each trial (Matlab 2016b) and trials were discarded if the RMS exceeded 20 µV. Trials in which GoRTs were < 300 ms or > 1280 ms were removed. Peak-to-peak MEP amplitudes in the right FDI muscle were calculated for each trial and outliers (± 3 SD) were removed. The MEP amplitudes were averaged across all trials per trial type, at each stimulation time point. Conditions of interest were early, go and stop (Figure 5D). For the stop condition, we only included MEPs of successful stop trials. Next, percentage SICI/LICI was calculated for each condition using the following formula: Inhibition % = [1 – (MEP_CS + TS_ / MEP_TS_)] * 100. Scores between 0% and 100% indicate inhibition while scores below 0% indicate facilitation. Normality of the data was visually inspected with Q-Q plots and did not hold for the LICI measures.

### Statistics

### *Behavioral data*

Statistical analysis were performed with the Statistical Package for the Social Sciences (IBM SPSS, Version 24.0, Armonk, NY, USA). First, statistical analyses were performed on the behavioral data that were collected at the beginning of each session, prior to stimulation. The effect of age group (young, older) and session (SICI, LICI) on P(inhibit) was analyzed with a 2-way repeated measures analysis of variance (ANOVA_RM_). To asses age-related differences in reactive inhibition, a 2 (age group) x 2 (session) ANOVA was performed with SSRT as dependent variable. Age-related differences in proactive inhibition were tested with a 2 (age group) x 2 (session) x 3 (SSP: 0, 20, 40) ANOVA_RM_ with GoRT as dependent variable. GoRTs were expected to increase as a function of SSP [27]. Second, we investigated the effects of age group and session on the participant-specific stop time and P(inhibit) during TMS using a two-way ANOVA_RM_.

### *TMS intensities, motor threshold and resting measures of CSE, SICI and LICI*

Age-related differences in TMS intensities, motor threshold and resting-measures of CSE and intracortical inhibition were investigated. The TS intensity and the size of the amplitude of the unconditioned MEPs in rest were tested with a 2 (age group) x 2 (session) ANOVA_RM_. The CS intensity in the SICI session, the tMT in the SICI session and SICI in rest were analyzed with student *t*-tests with age group as independent variable. Due to deviation from the normality assumption, the effect of age on LICI in rest was tested with the non-parametric Mann-Whitney *U*-test.

### Modulation of CSE and SICI during reactive inhibition

In the SICI session, we aimed to address the effect of age on SICI in successful stop relative to go trials. We expected a difference in SICI between stop and go in young [7], but not in older adults. The data of the 20 and 40% SSP task blocks (Figure 4) was used, and trials were averaged across the 20 and 40% SSP conditions. A 2-way ANOVA_RM_ with age group as between variable, condition (early, stop, go) as within variable, and SICI as dependent variable. Tests of the specified hypothesis were assessed within the 2-way ANOVA_RM_ with planned comparisons (i.e. stop versus go). The same ANOVA_RM_ was performed on the unconditioned MEPs to assess the modulation of CSE. The average size of the MEP amplitude in the TS alone trials in the rest block was added as a covariate in the SICI analysis since the size of the unconditioned MEP amplitude has been shown to influence SICI [42].

Associations between reactive inhibition and the modulation of SICI were investigated with Pearson correlation coefficients in young and older adults separately. More specifically, the difference in SICI between go and stop trials was correlated with the SSRT.

### *Modulation of CSE and LICI as a function of SSP*

The modulation of LICI as a function of SSP (indicated by the color of the bar) was tested with a 2-way ANOVA_RM_ with age group as between variable, SSP (0, 20, 40) as within variable, the unconditioned MEP amplitude in rest as covariate and LICI in the early condition as dependent variable. It was expected that the amount of LICI in the beginning of the trial would increase with increasing SSP. Because of deviation from the normality assumption, LICI was transformed using an exponential function. The same ANOVA_RM_ was performed on the unconditioned MEPs to assess the modulation of CSE.

Associations between proactive response slowing and the modulation of LICI were investigated with Spearman correlation coefficients in young and older adults separately. To get a single-value index of the modulation of LICI and GoRTs with increasing SSP, we calculated the slope with a simple linear regression (Matlab 2016b). The slopes were correlated to investigate the association between the modulation of LICI and response slowing.

The significance level was set at *p* < .05 for all tests. Greenhouse-Geisser correction was applied when the assumption of sphericity was violated. All post-hoc comparisons were conducted with Tukey HSD. All data are presented as mean ± standard deviation in the text.
